
JOURNAL INFORMATION
==============================
NLM Title Abbreviation: Wirtschaftsdienst
Journal ID: Wirtschaftsdienst
NLM Journal ID: 101086612

Wirtschaftsdienst (Hamburg, Germany : 1949)

ISSN: 0043-6275

ARTICLE INFORMATION
==============================
PMCID: PMC7813613
PMID: 33487773
DOI: 10.1007/s10273-021-2827-3
Article ID: 2827
Article version: 1
Subjects: Ökonomische Trends

Energienachfrage und CO2-Emissionen nach COVID-19

Energy demand and CO2 emissions according to COVID-19

Löschel Andreas andreas.loeschel@wiwi.uni-muenster.de

Werthschulte Madeline madeline.werthschulte@wiwi.uni-muenster.de

grid.5949.1 0000 0001 2172 9288 Westfälische Wilhelms-Universität Münster, Am Stadtgraben 9, 48143 Münster, Deutschland
Electronic publication date: 2021 Jan 19
Print publication date: 2021
Volume: 101
Issue: 1
First page: 64
Last page: 66
Copyright: © Der/die Autor(en) 2021
License: Open Access: Dieser Artikel wird unter der Creative Commons Namensnennung 4.0 International Lizenz (https://creativecommons.org/licenses/by/4.0/deed.de) veröffentlicht.
Open Access wird durch die ZBW - Leibniz-Informationszentrum Wirtschaft gefördert.
License URL: https://creativecommons.org/licenses/by/4.0/

issue-copyright-statement: © The Author(s) 2021

==============================
Prof. Dr. Andreas Löschel lehrt Mikroökonomik, insbesondere Energie- und Ressourcenökonomik, an der Westfälischen Wilhelms-Universität Münster und ist Research Associate am Leibniz-Zentrum für Europäische Wirtschaftsforschung Mannheim.

Das Jahr 2020 wurde maßgeblich von der COVID-19-Pandemie bestimmt. Neben den gesundheitlichen und gesamtwirtschaftlichen Konsequenzen hatte die Pandemie auch weitreichende energiewirtschaftliche Folgen. Durch die Lockdown-Beschränkungen und die „Stay-at-Home"-Aufforderungen kam es zu einer Reduzierung der globalen $CO_2$-Emissionen, der lokalen Luftschadstoffe sowie der Energienachfrage (Friedlingstein et al., 2020, Le Quéré et al., 2020, Liu et al,. 2020, Ou et al., 2020). Die globalen $CO_2$-Emissionen sind 2020 um etwa 7 % gesunken, in Europa um etwa 11 %. In der ersten Aprilhälfte, als die Einschränkungen insbesondere in den USA und Europa besonders umfassend waren, betrugen die Rückgänge gegenüber dem Vorjahr sogar etwa 17 %. Seit dem zweiten Weltkrieg hat es noch keinen solchen Rückgang der $CO_2$-Emissionen gegeben. Dabei sind durch die Einschränkungen vor allem die Emissionen im Verkehrssektor und die Ölnachfrage gesunken, aber auch die Industrieemissionen sowie die kohlebedingten Emissionen in der Stromerzeugung (Friedlingstein et al., 2020).

In Deutschland ist der Primärenergieverbrauch 2020 im Vergleich zu 2019 um etwa 9 % gesunken (BDEW, 2020a). In den von den Ausgangsbeschränkungen am stärksten betroffenen Monaten April und Mai 2020 ging der Stromverbrauch um 10 % bzw. 11 % im Vergleich zum Vorjahr (AGEB, 2020) zurück. In Kombination haben der Rückgang der Stromnachfrage aufgrund der Corona-Pandemie, die hohen $CO_2$-Preise im Europäischen Emissionshandelssystem seit Ende 2018 und günstige Verhältnisse für erneuerbare Energien zu einer deutlichen Verschiebung des Energiemix geführt. Die stärksten Rückgänge verzeichnen die Energieträger Braun- und Steinkohle, mit jeweils etwa 18 % weniger Verbrauch als 2019 (BDEW, 2020a). Im Gegenzug stammte mehr als die Hälfte der Nettostromerzeugung 2020 aus erneuerbaren Energien (Fraunhofer ISE, 2020). Dies bedeutet einen Rückgang der $CO_2$-Emissionen in der Energiewirtschaft von etwa 37 Mio. t $CO_2$ oder 15 % (BDEW, 2020a). Da diese Zahlen vorläufig sind, könnten die Auswirkungen des erneuten „harten" Lockdowns im Dezember sogar noch größere Rückgänge bewirkt haben. So wird insbesondere durch die Effekte der COVID-19-Pandemie in der Energiewirtschaft das deutsche Klimaziel für 2020, eine Minderung der klimaschädlichen Treibhausgase von 40 % gegenüber dem Vergleichsjahr 1990, erreicht – die Emissionen sinken um ca. 42 % (Agora Energiewende, 2021). Allerdings bedeutet dies kein gleichzeitiges Erfüllen der Energiewendeziele in allen Sektoren (Löschel et al., 2021a).

Die mittel- und langfristigen Auswirkungen der COVID-19-Pandemie auf Energieverbrauch sowie globale und lokale Schadstoffe sind ungewiss (Freire-González und Font Vivanco, 2020, Gillingham et al., 2020, Ou et al., 2020). Auf den letzten Rückgang der globalen $CO_2$-Emissionen infolge der Wirtschafts- und Finanzkrise 2009 folgte ein rasches Zurückspringen der Emissionen in den 2010er Jahren. Ein solches Zurückspringen der Emissionen kann nun verhindert werden, wenn durch die Maßnahmenpakete zur Abschwächung der wirtschaftlichen Folgen der COVID-19-Pandemie grüne Investitionen und klimafreundliches Verhalten nachhaltig etabliert werden (Cohen, 2020; European Commission, 2020; Hodges und Jackson, 2020; Pearson et al., 2020; Rosenbloom und Markard, 2020). Die Stimuli der Konjunkturprogramme dürfen aber grüne Investitionen nicht nur kurzfristig attraktiv machen. Vielmehr muss der klimapolitische Rahmen so gestaltet werden, dass sich das grundlegende Geschäftsmodell der Unternehmen und die Entscheidungen der Haushalte ändern. Dazu sind höhere $CO_2$-Preise das Instrument der Wahl, ergänzt durch einen nachhaltigen Infrastrukturausbau und die Förderung von Forschung und Entwicklung, Innovationen, Diffusion und Adoption neuer $CO_2$-armer Technologien (Löschel und Pittel, 2020). Eine Strompreisreform für Haushalte und Industrie mit höheren $CO_2$-Preisen und niedrigeren Strompreisen durch die Senkung der Umlagen, Abgaben und Steuern auf Elektrizität wird den aktuellen auch verteilungspolitischen Anforderungen besonders gerecht (Löschel et al., 2020).

Abbildung 1

**Energiespargewohnheiten (A) und Wahrscheinlichkeit, einzelne Maßnahmen zu ergreifen (B)**

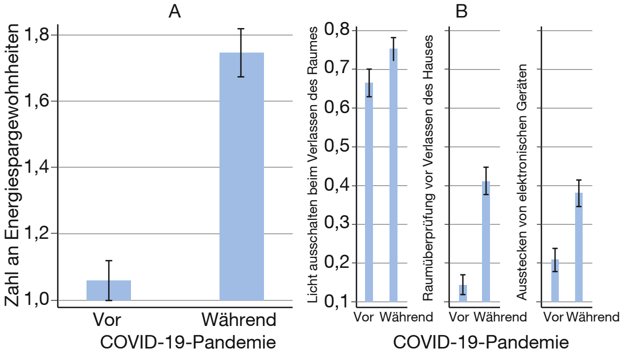

Quelle: Löschel et al. (2021b).

Abbildung 2

**Ausstecken von elektronischen Geräten und zuhause verbrachte Zeit während der COVID-19-Pandemie**

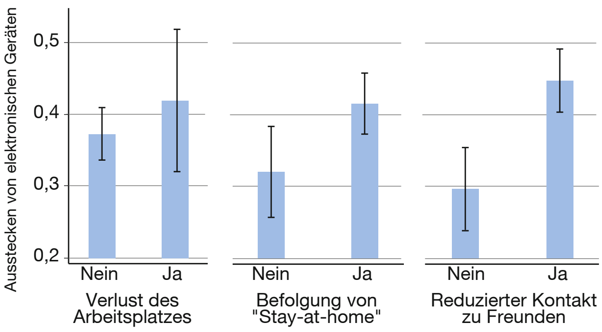

Quelle: Löschel et al. (2021b).

Gleichzeitig hängt der langfristige Übergang zu nachhaltigen Konsummustern entscheidend von Veränderungen im Verbraucherverhalten ab und davon, ob bzw. wie die Konsumgewohnheiten durch COVID-19 beeinflusst wurden. Inwieweit die COVID-19-Pandemie die Konsumgewohnheiten beeinflusst hat, soll am Beispiel des Stromverbrauchs dargestellt werden, der zu einem Viertel von der Nachfrage der Haushalte bestimmt wird (BDEW, 2020b). Dem deutschen Rückgang im Stromverbrauch 2020 steht ein leichter Anstieg der Stromnachfrage der privaten Haushalte um etwa 2 % entgegen (BDEW, 2020a). Aufgrund der COVID-19-Pandemie und der Ausgangsbeschränkungen waren Haushalte angehalten, mehr Zeit zuhause zu verbringen, weswegen der Stromverbrauch dieser Gruppe angestiegen ist. Es ist jedoch unklar, was die erhöhte Zeit zuhause für die Energieeffizienz und die langfristigen Verbrauchsmuster von Haushalten bedeutet. Möglicherweise haben sie aufgrund der Pandemie Energiespargewohnheiten entwickelt, sodass der tatsächliche Verbrauch ohne diese Veränderungen im Verbraucherverhalten noch höher wäre.

Es gibt mindestens zwei Gründe, warum die COVID-19-Pandemie die Haushalte veranlasst haben könnte, neue Gewohnheiten und Strategien zum Energiesparen zu entwickeln. *Erstens* führte die Pandemie bei vielen Personen zu Veränderungen im Beschäftigungsstatus und im Arbeitseinkommen. Da es einen positiven Zusammenhang zwischen Stromverbrauch und Einkommen gibt (für Deutschland: Schulte und Heindl, 2017), werden Einkommensbeschränkungen zu einer Reduzierung des Stromverbrauchs führen. *Zweitens* könnten Haushalte, entsprechend der Salienzmodelle aus der Verhaltensökonomik (Bordalo et al., 2013; Gabaix, 2019), durch die erhöhte Zeit im Haus die stromverbrauchenden Geräte und Handlungen stärker wahrnehmen. Der Stromverbrauch wird salienter, wodurch Haushalte auch eher zu Stromsparmaßnahmen greifen könnten. Sowohl Einkommenseffekte als auch die stärkere Wahrnehmung des Verbrauchs würden dann dazu führen, dass Haushalte ihren Stromverbrauch im Zuge der politischen Maßnahmen zur Eindämmung von Corona reduzieren.

Löschel et al. (2021b) untersuchen diesen Zusammenhang zwischen COVID-19 und Energiespargewohnheiten mit deutschen Umfragedaten, die in zwei Befragungswellen erhoben wurden. Die erste Befragung wurde Anfang 2018 durchgeführt, die zweite Befragung im Mai 2020, also während des Lockdowns und der „Stay-at-home"-Aufforderungen in Deutschland. Die Kernergebnisse von Löschel et al. (2021b) sind in Abbildung 1 und 2 dargestellt. Abbildung 1 zeigt, dass Haushalte während der Pandemie neue Gewohnheiten entwickelten, während Abbildung 2 nahelegt, dass Salienzeffekte im Gegensatz zu Einkommenseffekten der Haupttreiber der Gewohnheitsbildung waren.

Abbildung 1 (A) zeigt, dass die durchschnittliche Zahl an Energiespargewohnheiten bei den Befragten während der COVID-19-Pandemie um ca. 67 % höher ist als vor der Pandemie (zweiseitiger t-Test, $p = 0{,}0000$). Ein ähnlicher Unterschied zeigt sich in den jeweiligen Wahrscheinlichkeiten, eine bestimmte Energiesparmaßnahme regelmäßig durchzuführen (vgl. Abbildung 1 (B)). Im Vergleich zu den Werten aus der ersten Befragungswelle steigt die Wahrscheinlichkeit, dass eine Person angibt, das Licht auszuschalten, wenn sie einen Raum verlässt, um 8 Prozentpunkte; jeden Raum zu überprüfen, bevor sie das Haus verlässt, um 27 Prozentpunkte; und Geräte nach der Nutzung auszustecken, um 17 Prozentpunkte. Diese Anstiege sind hochsignifikant (zweiseitige t-Tests, $p = 0{,}0002$ für das Ausschalten des Lichts, $p = 0{,}0000$ für das Überprüfen jedes Raums, $p = 0{,}0000$ für das Ausstecken von Geräten).

Abbildung 2 untersucht beispielhaft für das Ausstecken von Geräten, ob eher Einkommens- oder Salienzeffekte diesen Anstieg begründen. Dazu werden die Teilnehmenden der zweiten Befragungswelle hinsichtlich ihrer Betroffenheit von dem Lockdown und den Politikmaßnahmen im Mai 2020 gruppiert. Demnach sind Teilnehmende, die nach Ausbruch der Pandemie mehr Zeit zuhause verbringen, eher bereit, elektronische Geräte nach Gebrauch auszustecken. Die Unterschiede sind jedoch nur bei denjenigen Konsument:innen signifikant, die angeben, dass sie aufgrund der „Stay-at-home"-Aufforderung oder eines reduzierten Kontakts zu Freunden oder Familie mehr Zeit zuhause verbringen (zweiseitige t-Tests, $p = 0,0173$ für stay-at-home, $p = 0,0001$ für reduzierten Kontakt zu Freunden oder Familie).

Dementgegen gibt es keinen signifikanten Unterschied in der Wahrscheinlichkeit, Geräte auszustecken, zwischen denjenigen, die ihren Arbeitsplatz aufgrund der Pandemie verloren haben, und denjenigen, deren Arbeitsplatz von der Pandemie nicht betroffen war (zweiseitiger t-Test, $p = 0,3938$). Dieser nicht signifikante Zusammenhang zwischen einem Beschäftigungsverlust und der Durchführung von Energiesparmaßnahmen ist inkonsistent mit Einkommenseffekten als Treiber des Anstiegs an Stromspargewohnheiten. Vielmehr scheint es so, als würden verhaltensbedingte Motive durch mehr Zeit, die zuhause verbracht wird, den Anstieg der Energiespargewohnheiten im Vergleich zum Niveau vor COVID-19 erklären.

Die COVID-19-Pandemie und die getroffenen Maßnahmen könnten also die Bildung von Energiespargewohnheiten unterstützt haben und so einen langfristigen Wandel hin zu nachhaltigen Konsum- und Produktionsmustern beschleunigt haben. Es bleibt offen, ob COVID-19 ähnliche langfristige Verhaltensänderungen auch in den anderen Sektoren hervorgerufen hat. Einige aktuelle Arbeiten deuten auf weitere Spill-Over-Effekte der COVID-19-Pandemie hin (Newbold et al., 2020; Helm, 2020). So könnten z. B. Gewohnheits- und Lerneffekte in Kombination mit den Fortschritten der Informations- und Kommunikationstechnologien dazu führen, dass auch langfristig Dienstreisen durch Videokonferenzen und Homeoffice ersetzt werden. Globale Lieferketten könnten verlagert werden durch einen Anstieg der Nachfrage nach im eigenen Land produzierten Gütern aufgrund der offensichtlich gewordenen Unsicherheit. Beide Faktoren würden einen nachhaltigen Rückgang der durch Transport und Güterverkehr verursachten Emissionen bewirken. Die Auswirkungen sind aber noch weitgehender, z. B. könnten Ausgehbeschränkungen zu einer erhöhten Wertschätzung von Naherholungsgebieten geführt haben und einer entsprechenden Bereitschaft, zu deren Erhalt beizutragen (Newbold et al., 2020; Helm, 2020). Um diese nachfrageseitigen Trends zu verstärken, sollten die erreichten Verhaltensanpassungen von politischer Seite durch höhere $CO_2$-Preise, nachhaltigen Infrastrukturausbau und die Förderung $CO_2$-armer Technologien unterstützt werden.


Madeline Werthschulte ist an beiden Instituten als Wissenschaftlerin beschäftigt.


Literatur

Agora Energiewende (2021), Die Energiewende im Corona-Jahr: Stand der Dinge 2020. Rückblick auf die wesentlichen Entwicklungen sowie Ausblick auf 2021, https://static.agora-energiewende.de/fileadmin2/Projekte/2021/2020_01_Jahresauswertung_2020/200_A-EW_Jahresauswertung_2020_WEB.pdf (8. Januar 2021).
AGEB (2020), Energieverbrauch in Deutschland, Daten für das 1. bis 3. Quartal 2020, https://ag-energiebilanzen.de/20-0-Berichte.html (29. Dezember 2020).
BDEW (2020a), Die Energieversorgung 2020 - Jahresbericht, https://www.bdew.de/media/documents/Jahresbericht_2020_20201218.pdf (29. Dezember 2020).
BDEW (2020b), Stromverbrauch in Deutschland nach Verbrauchergruppen 2019, https://www.bdew.de/service/daten-und-grafiken/nettostromverbrauch-nach-verbrauchergruppen (1. September 2020).
Bordalo P Gennaioli und A.^Shleifer N Salience and Consumer Choice Journal of Political Economy 2013 121 5 803 843 10.1086/673885
Cohen M J Does the COVID-19 outbreak mark the onset of a sustainable consumption transition? Sustainability: Science, Practice, and Policy 2020 16 1 1 3
European Commission (2020), Europe’s moment: Repair and prepare for the next generation, https://ec.europa.eu/commission/presscorner/detail/en/ip_20_940 (29. Dezember 2020).
Fraunhofer ISE (2020), Energy-Charts, https://energy-charts.info/charts/energy_pie/chart.htm?l=de&c=DE (1. Januar 2021).
Friedlingstein P O’Sullivan M Jones M W Andrew R M Hauck J Olsen A Global Carbon Budget 2020 Earth System Science Data 2020 12 4 3269 3340 10.5194/essd-12-3269-2020
Freire-González, J. und D. Font Vivanco (2020), Pandemics and the Environmental Rebound Effect: Reflections from COVID-19, Environmental and Resource Economics.10.1007/s10640-020-00448-7 PMC7346853 32836832
Gabaix, X. (2019), Behavioral inattention, in B. D. Bernheim, S. DellaVigna und D. Laibson (Hrsg.), Handbook of Behavioral Economics: Applications and Foundations 2, Bd. 2, Elsevier B. V.
Gillingham K T Knittel C R Li J Ovaere und M.^Reguant M The Short-run and Long-run Effects of Covid-19 on Energy and the Environment Joule 2020 4 7 1337 1341 10.1016/j.joule.2020.06.010 32835174 PMC7305502
Helm D The Environmental Impacts of the Coronavirus Environmental and Resource Economics 2020 76 1 21 38 10.1007/s10640-020-00426-z PMC7204192 32382212
Hodges K Jackson J Pandemics and the global environment Science Advances 2020 6 28 eabd1325 10.1126/sciadv.abd1325 32937548 PMC10662316
Le Quéré et al. (2020), Temporary reduction in daily global CO2 emissions during the COVID-19 forced confinement, Nature Climate Change, 1–8.
Liu F Abrupt decline in tropospheric nitrogen dioxide over China after the outbreak of COVID-19 Science Advances 2020 6 28 eabc2992 10.1126/sciadv.abc2992 32923601 PMC7455481
Löschel, A., V. Grimm, B. Lenz und F. Staiß (2020), Klimaschutz vorantreiben, Wohlstand stärken - Kommentierung zentraler Handlungsfelder der deutschen Energiewende im europäischen Kontext, Expertenkommission „Energie der Zukunft“.
Löschel, A., V. Grimm, B. Lenz und F. Staiß (2021a), Stellungnahme zum achten Monitoring-Bericht der Bundesregierung für die Berichtsjahre 2018 und 2019, Expertenkommission „Energie der Zukunft“.
Löschel A Pittel K Der EU Green Deal und deutsche Anstrengungen zum Klimaschutz in der Coronakrise ifo Schnelldienst 2020 6 73 6 9
Löschel, A., M. Price, L. Razzolini und M. Werthschulte (2021b), Implications of the COVID-19 pandemic for household energy consumption: The stay-at-home orders and energy conservation habits, mimeo.
Newbold S C Finnoff D Thunström L Ashworth und J. F.^Shogren M Effects of Physical Distancing to Control COVID-19 on Public Health, the Economy, and the Environment Environmental and Resource Economics 2020 76 4 705 729 10.1007/s10640-020-00440-1 PMC7399603 32836854
Ou, S. et al. (2020), Machine learning model to project the impact of COVID-19 on US motor gasoline demand, Nature Energy, (September).10.1038/s41560-020-00711-7 PMC7543031 33052987
Pearson R M Sievers M McClure E C Turschwell und R. M.^Connolly M P COVID-19 recovery can benefit biodiversity Science 2020 368 6493 838 839 10.1126/science.abc1430 32439784
Rosenbloom D Markard J A COVID-19 recovery for climate Science 2020 368 6490 447 10.1126/science.abc4887 32355005
Schulte I Heindl P Price and income elasticities of residential energy demand in Germany Energy Policy 2017 102 512 528 10.1016/j.enpol.2016.12.055
